# MIA-Sig: multiplex chromatin interaction analysis by signal processing and statistical algorithms

**DOI:** 10.1186/s13059-019-1868-z

**Published:** 2019-11-25

**Authors:** Minji Kim, Meizhen Zheng, Simon Zhongyuan Tian, Byoungkoo Lee, Jeffrey H. Chuang, Yijun Ruan

**Affiliations:** 1 The Jackson Laboratory for Genomic Medicine, Farmington, CT USA; 2 Department of Genetics and Genome Sciences, University of Connecticut Health Center, Farmington, CT USA

**Keywords:** 3D genomics, Multiplex chromatin interactions, ChIA-Drop, Signal processing, Algorithms

## Abstract

The single-molecule multiplex chromatin interaction data are generated by emerging 3D genome mapping technologies such as GAM, SPRITE, and ChIA-Drop. These datasets provide insights into high-dimensional chromatin organization, yet introduce new computational challenges. Thus, we developed MIA-Sig, an algorithmic solution based on signal processing and information theory. We demonstrate its ability to de-noise the multiplex data, assess the statistical significance of chromatin complexes, and identify topological domains and frequent inter-domain contacts. On chromatin immunoprecipitation (ChIP)-enriched data, MIA-Sig can clearly distinguish the protein-associated interactions from the non-specific topological domains. Together, MIA-Sig represents a novel algorithmic framework for multiplex chromatin interaction analysis.

## Background

Traditional 3D genome mapping efforts have suggested complex chromosomal folding structures. In particular, methods based on high-throughput sequencing capture bulk chromatin contacts (Hi-C; Lieberman-Aiden et al. [18]) or enrich for chromatin contacts involving a specific protein (ChIA-PET; Fullwood et al. [9]). Both of these methods rely on proximity ligation and therefore can only reveal population averages of pairwise contacts. Thus, they lacked the ability to simultaneously capture multiple loci involved in a chromatin complex in an individual cell.

To overcome these drawbacks, novel experimental methods have recently been developed to capture multiplex chromatin contacts with single-molecule resolution. For instance, GAM (Beagrie et al. [2]) identifies multi-way interactions by capturing multiple DNA elements co-existing in a given nuclear slice, SPRITE (Quinodoz et al. [25]) barcodes individual chromatin complexes via a split-pool strategy, and ChIA-Drop (Zheng et al. [31]) partitions each complex into a microfluidic droplet for barcoding and amplification. Collectively, these emerging 3D genome mapping technologies are advancing the frontier of the nuclear architecture field. However, as with other genomic approaches prone to the background noise, the noisy and high-dimensional nature of the multiplex data poses unique computational challenges that cannot be readily addressed with existing tools that are tailored for pairwise interactions data.

Numerous software tools are available for analyzing data generated by genome-wide 3D architecture assays such as 3C, 4C, 5C, and the most common assay Hi-C. For example, HiCNorm (Hu et al. [10]) and Hi-Corrector (Li et al. [17]) explicitly or implicitly correct the bias observed in Hi-C data. Fit-Hi-C (Ay et al. [1]) and GOTHiC (Mifsud et al. [20]) aim to assess the statistical significance of intra-chromosomal contacts by incorporating bias in the background null model. The authors of Fit-Hi-C emphasized the importance of accurately modeling the inverse relationship between genomic distance and contact probability. Similarly, multiplex data also depend on the distance, but currently available tools cannot be naively applied since (1) genomic distance is now multi-dimensional instead of 1D, i.e., a complex with *n*-way contacts yield *n* − 1 neighboring distances and $\left(\genfrac{}{}{0pt}{}{n}{2}\right)=\frac{n\left(n-1\right)}{2}$ pairwise distances, and (2) contact probability must be defined for all *n*-way contacts, yet it is unclear if ten-way contact is as likely as two-way contact.

Another crucial component in Hi-C data analysis is to call topologically associating domains (TADs), loosely defined as regions with more contacts inside than outside. In general, TADs appear as squares along the diagonal in the contact map, and the goal is to identify and segment the genome. There are more than 20 TAD calling algorithms (Zufferey et al. [32]), some of which convert the contact map into a 1D signal along the diagonal for subsequent segmentation or into a graph and apply community detection algorithms. To run the existing tools, multiplex data must first be converted into a contact map. However, enumerating over all possible pairs in a complex is computationally expensive and may introduce additional bias since the number of pairwise interactions increases quadratic in *n*. In other words, a complex with 5 fragments yields 28 pairs instead of 1 pair for a complex with 2 fragments. This approach would also lose valuable multiplexity information.

Conventional studies focused on interactions within these TADs identified computationally. However, a recent Hi-C study has suggested that multiple TADs can interact with each other to accommodate molecular functions during the development (Paulsen et al. [22]). The authors inferred confident domain-wise interactions by finding cliques in a graph, where nodes represent TADs and edges are contact frequency between TADs. Unlike Hi-C datasets, the multiplex data naturally provide interactions among any number of TADs. Thus, it is desirable to exploit this information and assess the statistical significance of these observed inter-TAD interactions.

In parallel, algorithms have been developed to analyze protein-enriched 3D architecture data from assays such as ChIA-PET. Similar to Hi-C, ChIA-PET data are also prone to bias and noise, which are computationally filtered out by statistical algorithms such as ChIA-PET tool (Li et al. [16]) and chiasig (Paulsen et al. [23]). The main idea is to model interaction frequency between two loci as hypergeometric distribution or the non-central hypergeometric distribution. To accommodate recently developed variants HiChIP (Mumbach et al. [21]) and PLAC-seq (Fang et al. [8]), researchers developed hichipper (Lareau and Aryee [15]), fithichip (Bhattacharyya et al. [4]), and MAPS (Juric et al. [11]) to remove systematic biases and identify significant loops. In ChIA-PIPE (Capurso et al. [5]), the de-noising is done by filtering out loops without peak supports in the anchors. Unfortunately, these tools are specifically designed to model interactions between two loci and would not readily generalize to those involving more than two loci.

Thus, to fill in the gap in novel software for analyzing multiplex data, we developed MIA-Sig (*M*ultiplex *I*nteractions *A*nalysis by *Sig*nal processing algorithms) with a set of Python modules tailored for ChIA-Drop and related data types. MIA-Sig has the following components: (1) calling statistically significant complexes and removing experimental noise, (2) calling TADs on multiplex data, and (3) identifying meaningful multi-way inter-TAD contacts.

## Results

### Distance test resolves multiplets and removes experimental noise

**Fig. 1:**
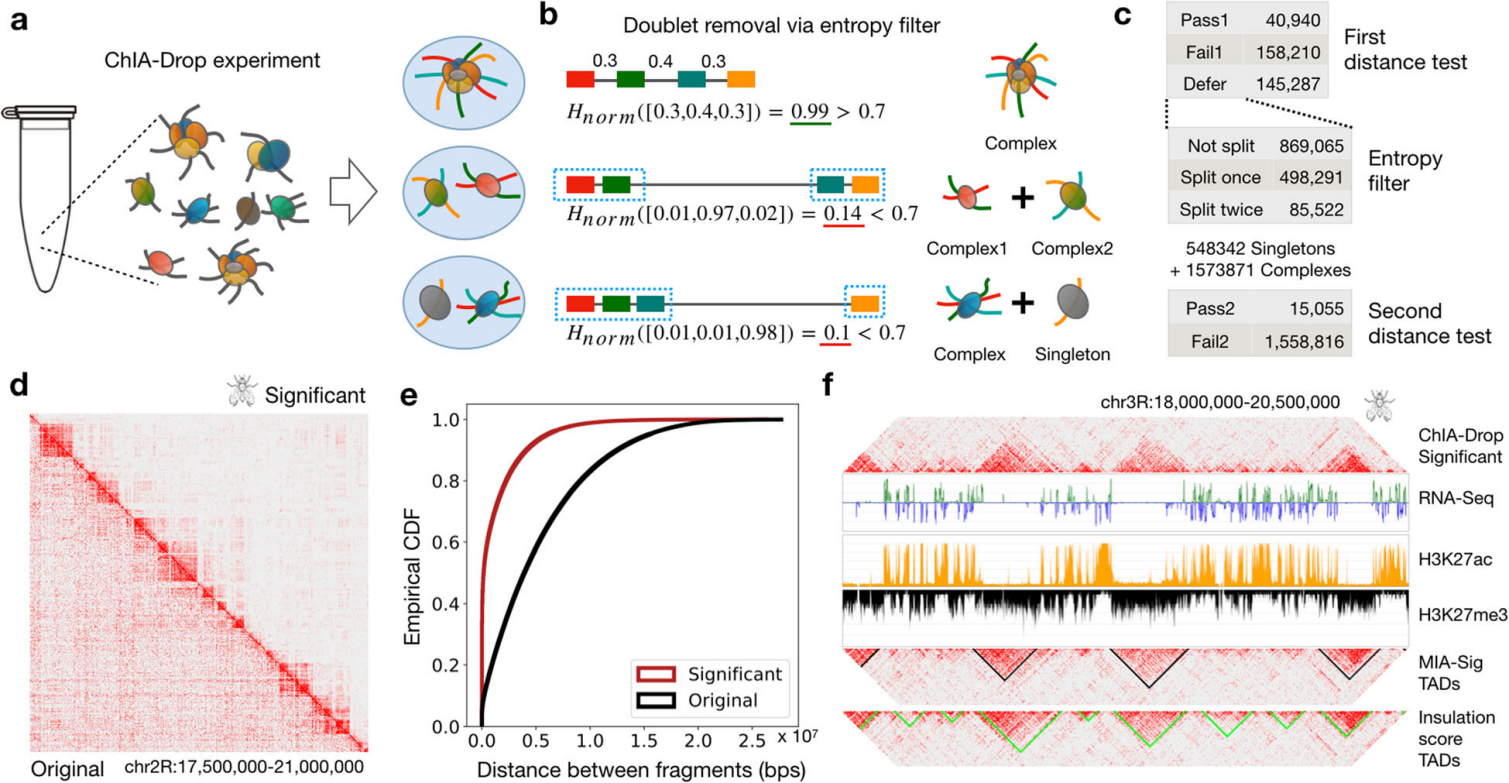
Performance of MIA-Sig on *Drosophila* S2 cells ChIA-Drop data. **a** ChIA-Drop experiments are designed to encapsulate each chromatin complex in a droplet, but the encapsulation is a random process and sometimes results in more than one complex in a droplet (multiplets). **b** MIA-Sig aims to detect multiplets by computing the normalized Shannon entropy *H*_norm_ (the “Methods” section). It separates a complex at the largest distance if *H*_norm_ is smaller than a threshold, which is 0.7 in this example. This threshold is determined from the normalized Shannon entropy of the expected null model. **c** Summary statistics of the distance test indicate that the entropy filter resolves around 500,000 doublets and 85,000 triplets, from which 15,055 complexes pass the second distance test. **d** 2D heatmap comparison of original (bottom triangle) and significant (upper triangle) complexes demonstrates that MIA-Sig removes off-diagonal noise. **e** Empirical cumulative distribution function for the neighboring distances of original and significant complexes (two-sided Kolmogorov-Smirnov test statistic =0.47, *p* value <2.2 × 10^−16^ ). **f** Comparison of TADs called from significant putative complexes (MIA-Sig) and from enumerating all pairs of fragments (insulation score). MIA-Sig more specifically separates active regions (high H3K27ac and low H3K27me3) rather than assigning them to TADs.

A central challenge in ChIA-Drop data analysis is to distinguish the true biological chromatin complexes from the experimental noise. One possible source of noise is an event that two or more chromatin complexes are potentially encapsulated in the same microfluidic droplet and then are assigned the same barcode, yielding a multiplet (Fig. 1a). The problem also prevails in microfluidic-based single-cell RNA-seq data, which is then resolved computationally via dimensionality reduction and clustering (Wolock et al. [30]). However, methods developed for single-cell transcriptomics data are not apt for multiplex chromatin interactions data since (1) the signal for chromatin interactions is point data (fragment is captured or not captured) rather than continuously valued data (gene expression level), and (2) multiplex chromatin interaction data are inherently more sparse than the single-cell transcriptomics data, due to the lack of cell barcodes.

Therefore, we devised a distance test with an entropy filter based on the biological knowledge that most meaningful chromatin interactions occur in a certain distance range, while those outside the range are likely noise (Lajoie et al. [14]). By converting the distances between fragments into a probability vector, we compute the normalized Shannon entropy (Shannon [27]), ranging from 0 to 1. If a droplet contains a single complex, the fragments are presumably close and equally spaced, resulting in high entropy close to 1. In the case of a doublet, two independent complexes would be separated by a single large distance, resulting in low entropy close to 0, which can then be separated into two singlets (Fig. 1b). The cutoff threshold is determined by the average normalized Shannon entropy of the expected null distribution as described below.

To identify significant chromatin complexes, a resampling-based distance test is applied before and after the entropy filter (Fig. 1c; Additional file 1: Figure S1a; the “Methods” section). We verify that the distance distribution of expected complexes from resampling (computational null) and that of pure DNA complexes (experimental null) are comparable, with the majority greater than 1 Mbps (Additional file 1: Figure S1b). Finally, we retained 55,995 statistically significant complexes in the *Drosophila* S2 ChIA-Drop data out of 3,075,926 putative complexes (Additional file 1: Figure S1c). Filtering to retain significant complexes preserves the TADs along the diagonal of the 2D heat maps, while reducing the off-diagonal noise (Fig. 1d; visualization through Juicebox (Durand et al. [7])). A shift in distance distributions from large (original) to small (significant) supports that meaningful interactions are captured within 10 kb and 1 Mb, mostly from complexes with 5 or more fragments (Fig. 1e; Additional file 1: Figure S2).

Of the significant chromatin complexes, 15,055 (27%) were from the entropy filtering step that resolved doublets and triplets (Additional file 1: Figure S3a,b). For example, of complexes with 3 fragments (in *F*_3_), 499,613 are identified as “singlets” due to high entropy, and 284,540 are considered to be “doublets” due to low entropy. A general trend is that entropy is highest for those without any splits, lowest for a doublet with a singleton, and increases as the size of sub-complexes balance to be roughly equal.

Several parameters are fixed or to be chosen in the distance test. As mentioned earlier, the cutoff threshold in the entropy filter is computed for each fragment class based on the null distribution; for reference, some of the values used in this study are summarized in Additional file 1: Figure S3c. In general, the threshold is higher for the class with a high number of fragments than for that with a low number of fragments. Other parameters are to be chosen by the users: false discovery rate (FDR), ratio threshold (ratiothresh) for separating the second largest distance in the entropy test, and the sample size for constructing the null. We benchmarked a few values for some of these parameters and evaluated their effects by recording the number of significant complexes and by performing the two-sided K-S test on fragment-to-fragment distances of the original and significant complexes. As expected, the setting with a lower number of significant complexes had higher K-S statistics, likely because MIA-Sig kept a small portion of the highly confident complexes. Given the same FDR, a ratiothresh of 5 yields more complexes in the significant category and a slightly higher K-S statistics than a ratiothresh of 2. The current default parameters are FDR = 0.1 and ratiothresh = 2, but a more systematic evaluation of “real complexes” will be desirable in the future as more multiplex datasets become available.

### Wavelet-based segmentation method identifies TADs overlapping inactive regions

From the significant complexes, it is desirable to automatically call TADs for downstream analyses. Many TAD calling algorithms exist for Hi-C data (Zufferey et al. [32]), yet all are based on pairwise contacts. To retain multiplexity information, we developed an algorithm to call TADs directly from the ChIA-Drop data (the “Methods” section). The idea is to convert complexes into 1D signal track then apply wavelet transformation (Mallat [19]) to smooth the signal while retaining clear change points (Additional file 1: Figure S4a). This approach allows us to identify clear gaps between TADs, rather than segmenting the genome into consecutive TAD regions (Additional file 1: Figure S4b). MIA-Sig called 335 TADs with a wider range of sizes than 513 TADs called by pairwise “insulation score” (InS) approach; similarly, the gap sizes spanned a wider range for MIA-Sig TADs than for InS TADs (Additional file 1: Figure S5). Compared to InS TADs, the MIA-Sig TADs are less likely to overlap active regions characterized by high H3K27ac and low H3K27me3 (Fig. 1f), which are known to be the gaps between TADs in *Drosophila* (Rowley et al. [26]). This pattern is observed genome-wide: MIA-Sig TADs have a higher inactive mark (H3K27me3) than InS TADs, and MIA-Sig gaps have a higher active mark (H3K27ac) than InS gaps (Additional file 1: Figure S6).

### Binomial test detects frequent interactions among two or more TADs

Most interactions occur within a single TAD, but 23% of significant complexes also cross two or more TADs (Additional file 1: Figure S7a), consistent with previous findings (Paulsen et al. [22]). Thus, we identified frequent interactions involving multiple TADs by counting the occurrences and performing a binomial test (Additional file 1: Figure S7b; the “Methods” section). A set of TADs with frequent contacts are ultimately assigned low *p* values (Additional file 1: Figure S7), which can guide the researchers to perform validation experiments.

### Enrichment test retains strong interactions involving promoters

**Fig. 2:**
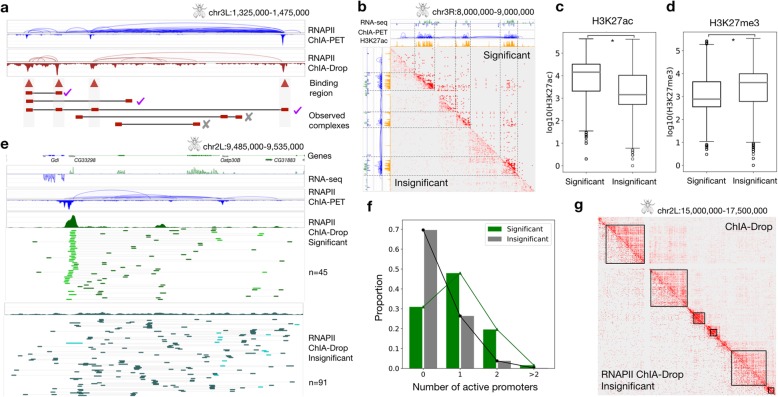
Enrichment test on *Drosophila* S2 cells RNAPII ChIA-Drop data. **a** MIA-Sig performs an enrichment test on RNAPII-enriched ChIA-Drop data by retaining complexes with fragments in strong binding regions, which also correspond to RNAPII ChIA-PET peaks. **b** The significant complexes are pronounced in regions with a high level of transcription, abundant loops, and active histone mark; insignificant complexes tend to be in inactive regions. **c** Log of H3K27ac signal for fragments in significant and insignificant complexes (one-sided Mann-Whitney *U* test, *p* value <2.2 × 10^−16^). **d** Log of H3K27me3 signal for fragments in significant and insignificant complexes (one-sided Mann-Whitney *U* test, *p* value <2.2 × 10^−16^). **e** Fragment coverage profile of significant complexes is similar to that of RNAPII ChIA-PET, with 45 promoter-centric multiplex interactions (green, non-promoters; light green, promoters). By contrast, insignificant complexes do not show any strong binding peaks in coverage, and 91 multiplex interactions are non-specific (turquoise, non-promoters; light turquoise, promoters). **f** Genome-wide, significant complexes have a higher proportion of active promoter fragments than insignificant complexes do (two-sided K-S test statistic =0.39, *p* value <2.2 × 10^−16^). **g** The insignificant RNAPII ChIA-Drop complexes from the enrichment test are comparable to the significant ChIA-Drop complexes from the distance test. TADs (black lines) are called by MIA-Sig on the latter complexes

Similar to ChIA-PET, ChIA-Drop can also enrich chromatin complexes involving a specific protein, such as RNAPII or CTCF. We implemented an enrichment test to estimate the significance of binding intensity of observed chromatin complexes and retain those with high binding intensity (Fig. 2a; the “Methods” section). An empirical null distribution is generated by placing the observed complex on a random location in the chromosome and recording the binding intensity. We verified that the empirical null and observed distributions differ significantly, with observed shifted to the right of the null (Additional file 1: Figure S8c,d). After the enrichment test, we retain 190,226 significant complexes out of 769,803 complexes (Additional file 1: Figure S8).

These significant complexes have their fragments in highly enriched domains characterized by high RNA-seq expression and H3K27ac signal with abundant RNAPII ChIA-PET loops (Fig. 2b). Genome-wide patterns confirm that significant complexes are biased towards active regions, whereas insignificant complexes are not (Additional file 1: Figure S9). Moreover, significant complexes have higher median H3K27ac signals and lower median H3K27me3 signals than insignificant complexes (Fig. 2c, d). A detailed view around a few genes shows that significant complexes are more likely to retain promoter-centric interactions than insignificant complexes (Fig. 2d; visualization through ChIA-View (Tian et al. [29])). This pattern is prevalent genome-wide, with 69% of significant complexes containing at least one promoter compared to only 30% of insignificant complexes (Fig. 2f). Notably, significant complexes are most likely to capture one active promoter and one or more non-promoters—possibly enhancers—while insignificant complexes are prone to detect interactions among non-promoters (Additional file 1: Figure S10). Among the promoter-involving fragments, those in significant complexes have higher median gene expression than those in insignificant ones.

### Insignificant RNAPII ChIA-Drop complexes emulate non-enriched ChIA-Drop data

As with many experimental protocols, the chromatin immunoprecipitation step is not 100% efficient and typically yields a 20–40% efficiency rate (Tang et al. [28]). Thus, we take advantage of the fact that enriched ChIA-Drop datasets also contain some background signal for chromatin complexes that did not specifically involve the protein of interest, similar to non-enriched ChIA-Drop data. Through the MIA-Sig enrichment test on RNAPII ChIA-Drop data, we can extract the non-enriched complexes from the insignificant complexes, which approximately emulate the ChIA-Drop data (Fig. 2g).

### Distance test can be applied to SPRITE data

We have developed MIA-Sig on ChIA-Drop and RNAPII ChIA-Drop data, but it could also be applied for de-noising multiplex chromatin interactions from other methods, such as SPRITE and GAM.

SPRITE uses three to five rounds of split-and-pool approach to barcode each chromatin complex by combinatorial indexing, with a theoretical assumption that many rounds of splitting and pooling should result in one unique barcode combination per chromatin complex. However, in practice, the split-and-pool process is limited to four to five rounds with a limited set of distinct barcodes, and in each round, potentially hundreds of thousands of chromatin complexes are assigned the same DNA oligo barcode. As a result, there is a certain non-zero probability of multiple complexes receiving an identical barcode combination. These unrelated complexes would be considered technical noise of SPRITE technique, which is somewhat similar to that of ChIA-Drop of unrelated complexes partitioned in the same microfluidic droplet.

**Fig. 3:**
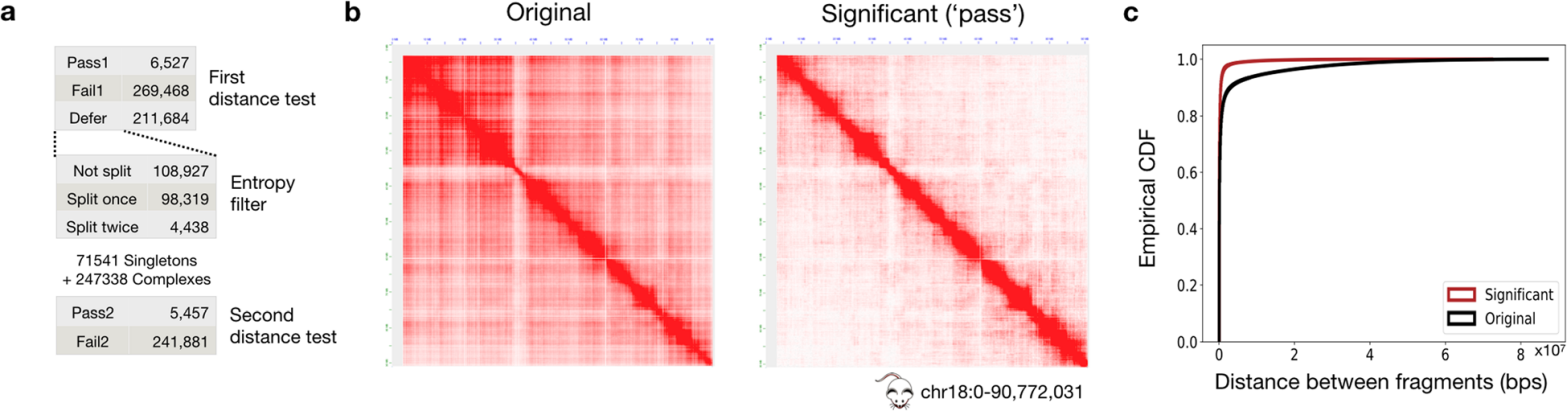
Distance test results on SPRITE mouse embryonic stem cell dataset. **a** Of 487,679 complexes in chr18, 11,984 (2.46%) complexes pass by the 2 distance tests. **b** A chromosome-wide heatmap is generated for original and significant complexes by enumerating all pairs of fragments. **c** Empirical cumulative distributive function (ECDF) for the neighboring distances of original and significant complexes (two-sided Kolmogorov-Smirnov test statistic =0.18, *p* value <2.2 × 10^−16^ )

As a proof-of-concept, we demonstrate the utility of MIA-Sig by performing the distance test on SPRITE data (Quinodoz et al. [25]) generated from F121 mouse embryonic stem cells (GSE114242). The data are pre-processed to convert reads into fragments of certain sizes and distances, and we selected intra-chromosomal complexes in chr18 (the “Methods” section). From the original 487,679 complexes, 11,984 complexes are identified as significant by the 2 distance tests preceding and following the entropy filter (Fig. 3a). The 2D contact maps of original complexes exhibit off-diagonal noise, whereas that of the significant complexes have the majority of the signal along the diagonal (Fig. 3b). We plot the empirical cumulative distribution of the fragment-to-fragment distances of original and significant complexes and observe that significant complexes have shorter distances than original complexes (Fig. 3c; two-sided Kolmogorov-Smirnov test statistic = 0.18, *p* value <2.2 × 10^−16^). These results indicate that MIA-Sig can indeed assess the statistical significance of complexes captured by SPRITE.

## Discussion

Many tools exist for analyzing traditional proximity ligation-based chromatin interaction data, such as Hi-C and ChIA-PET. By contrast, there is a lack of tools to comprehend the data generated by the recently developed multiplex interaction mapping techniques. To fill in this gap, we have developed MIA-Sig that is specifically designed to analyze multiplex chromatin interaction data.

The most significant functionality of MIA-Sig is to de-noise and identify statistically confident multiplex chromatin complexes in both non-enriched data and protein-enriched data. We applied an entropy concept from information theory to identify multiplets in ChIA-Drop and SPRITE data and implemented a simple yet relatively efficient method to evaluate the enrichment score of each complex in RNAPII ChIA-Drop data. In addition, we proposed a wavelet-based algorithm to call TADs on multiplex data. A unique feature of this approach is the ability to clearly distinguish TADs from gaps, which is of biological relevance in *Drosophila* samples. In particular, it is shown that TADs and gaps interleave in *Drosophila*, unlike in human or mouse where gaps are not as critical as they are in *Drosophila* (Rowley et al. [26]). These TADs merely serve as a unit in the downstream analysis, where we investigate the occurrence of simultaneous interactions among two or more TADs through the binomial test. A recent study support that these occurrences are important during development (Paulsen et al. [22]). New algorithmic ideas in this work are implemented in a publicly available package, along with scripts to generate data QC plots. Hence, MIA-Sig serves as a comprehensive pipeline including both data quality control and data analysis.

Although potentially a useful package, MIA-Sig nonetheless has its own drawbacks. One key assumption in the distance test is that a fragment far from the other fragments is likely a droplet contamination resulting in a doublet, a behavior yet to be confirmed experimentally and statistically. As with other TAD calling algorithms for Hi-C data, MIA-Sig’s TAD caller requires a set of parameters such as wavelet level and window size. We provide recommended parameters (Lajoie et al. [14]) for each representative model organism, but have not thoroughly tested due to lack of datasets. A critical pitfall in the inter-TAD binomial test is that we do not normalize the TAD interaction frequency by distance and size. In other words, we expect the closer and larger TADs to interact more frequently than others. Finally, in performing the enrichment test for RNAPII ChIA-Drop data, we do not use a background distribution model and instead draw an empirical null distribution via random sampling. A disadvantage of this approach is the computational cost, which can be demanding for large human datasets.

In sum, all multiplex chromatin interaction data could have a significant level of noise, and the principle nature of the noises is conceptually similar. The algorithm used in MIA-Sig considers general issues that should be applicable to all multiplex data. Although the current version of MIA-Sig is specifically developed based on the ChIA-Drop data, we demonstrated its capability to assess the significance of multiplex chromatin complexes in SPRITE data. With further modification and improvement, MIA-Sig should be directly applicable to any multiplex chromatin interaction data and also allow us to fully characterize similarity and differences between experimental protocols.

## Conclusions

**Fig. 4:**
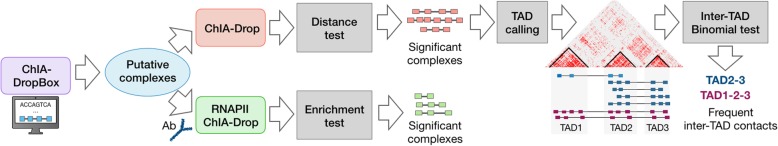
Summary of MIA-Sig algorithm. ChIA-Drop putative complexes from ChIA-DropBox pipeline are inputs to the distance test, which assigns *p* values to each complex to quantify its significance. Refined complexes enable TAD calling directly on the multiplex data. A binomial test identifies frequent contacts among multiple TADs. RNAPII-enriched ChIA-Drop putative complexes are assigned significance according to the level of enrichment

As we enter the era of single-cell and single-molecule 3D genome mapping, it will be imperative to develop algorithms to analyze data from these novel experimental protocols. We have presented an approach to solve the imminent problem of extracting statistically significant complexes from noisy signals, calling TADs, and identifying frequent inter-TAD contacts (Fig. 4). In addition, we offer a practical strategy to extract non-enriched ChIA-Drop from RNAPII ChIA-Drop.

We envision that MIA-Sig will be broadly applicable to any type of multiplex chromatin interaction data ranging from ChIA-Drop and SPRITE to GAM, under the aforementioned assumptions and with modifications. Here, we focused on the *Drosophila* ChIA-Drop and RNAPII ChIA-Drop data as a proof of concept and demonstrated that MIA-Sig filters and retains only the highly informative complexes and tested its applicability to the mammalian data generated by SPRITE. Finally, as a publicly available software package, MIA-Sig provides a valuable algorithmic framework for multiplex chromatin interaction data to be utilized by the broader scientific community.

## Methods

### Notation

An input dataset contains a set of chromatin complexes, each with two or more fragments. Let *OC*_*m*_ be the set of fragments contained in the *m*th “observed complex” (OC), for *m* ∈ {1, 2, …, *M*}, and *n* = |*OC*_*m*_| is the size of the set denoting the number of fragments in a complex. Each fragment *u* is subscripted by the complex index and superscripted by the fragment index and encodes the genomic location of its origin expressed as a triplet of chromosome, start and end positions. The distance *d* between fragments ${u}_m^a$ and ${u}_m^b$ is start(${u}_m^b$) − end(${u}_m^a$), and neighboring (fragment-to-fragment; F2F) distances are encoded in a vector

$$ {\mathbf{x}}_{\mathrm{F}2\mathrm{F}}\left({\mathrm{OC}}_m\right)=\left[d\left({u}_m^1,{u}_m^2\right),d\left({u}_m^2,{u}_m^3\right),\dots, d\left({u}_m^{n-1},{u}_m^n\right)\right], $$

and the total distance is *d*_tot_(OC_*m*_) =  ∑ **x**_F2F_(OC_*m*_); the probability vector ${\mathbf{p}}_{\mathrm{F}2\mathrm{F}}\left({\mathrm{OC}}_m\right)=\frac{{\mathbf{x}}_{\mathrm{F}2\mathrm{F}}\left({\mathrm{OC}}_m\right)}{d_{\mathrm{tot}}\left({\mathrm{OC}}_m\right)}$. For example, if an eighth complex ${OC}_8=\left\{{u}_8^1,{u}_8^2,{u}_8^3\right\}$ contains three fragments (chr2L, 100, 500), (chr2L, 1000, 1500), and (chr2L, 6000, 6500), then **x**_F2F_(OC_8_) = [500, 4500], *d*_tot_(OC_8_) = 5000, and ${\mathbf{p}}_{\mathrm{F}2\mathrm{F}}\left({\mathrm{OC}}_m\right)=\left[\frac{1}{10},\frac{9}{10}\right]$. Finally, we can partition *M* complexes OC_1_, OC_2_, …, OC_*M*_ into *F*_*j*_, where *j* is the number of fragments in a complex (OC_8_ belongs to *F*_3_ since it has three fragments).

### Distance test for non-enriched multiplex chromatin interactions data

#### Empirical null distribution and first distance test

Assuming that complexes are independent of chromosome, we perform the distance test separately for each chromosome. Motivated by the fact that each fragment class *F*_*j*_ has distinct distributions in F2F distances, we construct the expected null background distribution by randomly rewiring fragments. Specifically, all neighboring distances **x**_F2F_(OC_*m*_) for *m* ∈ {1, 2, …, *M*} are placed in a bucket *B*. For each observed *F*_*j*_, we randomly draw *j* − 1 elements (with replacement) from *B* to create 100,000 “expected complexes” (EC) ${\mathrm{EC}}_k^j$ for *k* ∈ {1, 2, …, 100,000} and store them in *F*_*j*_′. Note that since we only care about the distance between fragments, we can assume that every fragment starts at (chr, 1, 500) and each fragment is of equal length. In practice, we store minimum information to save compute memory (implementation details below). For each OC_*m*_ in *F*_*j*_, we compare its total F2F distance to total F2F distance in ${F}_j^{\prime }$ and record the proportion of expected complexes that have shorter distances than the observed complexes as the estimated “raw *p* value.” Formally, for a OC_*m*_ ∈ *F*_*j*_,

$$ \mathrm{pva}{\mathrm{l}}_{\mathrm{raw}}\left({\mathrm{OC}}_m\right)={\sum}_{k=1}^{\mathrm{100,000}}{1}_{\left\{{d}_{\mathrm{tot}}\left(\mathrm{O}{\mathrm{C}}_m\right)>{d}_{\mathrm{tot}}\left(\mathrm{E}{\mathrm{C}}_k^j\right)\right\}}, $$

where 1_{∗}_ is an indicator function. Assuming that complexes in each fragment class are independent, we subsequently separate the raw *p* values by *F*_*j*_ and adjust them for multiple hypothesis testing using Benjamini-Hochberg method (Benjamini and Hochberg [3]) with false discovery rate (FDR) of 0.1. The complexes with adjusted *p* value ≤0.1 are considered to be statistically significant and are classified as “pass1” (*F*_*j*, pass1_). Of those insignificant complexes with adjusted *p* value > 0.1, we “fail1” (*F*_*j*, fail1_) those with two fragments (OC_*m*_ ∈ *F*_2_ with adjusted *p* value > 0.1) and treat others in a separate category called “defer” (*F*_*j*, def_). These “deferred” complexes are passed onto the entropy filter to correct for droplet contamination.

#### Entropy filter

Some complexes in the “deferred” category may be due to the experimental noise that can be computationally detected. Specifically, this step aims to computationally correct for the undesired phenomenon of a droplet containing more than one chromatin complex (referred to as “doublet” for two, “triplet” for three, and “multiplet” for two or more). In single-cell RNA-seq (scRNA-seq; single-cell transcriptome) experiments, the outcome of a doublet would be a vector of real numbers indicating average expression of the two cells. By contrast, ChIA-Drop data only provide binary values indicating if a fragment was captured or not, with a variable number of fragments. Therefore, the effect of two complexes accidentally being encapsulated in a single droplet would be a large distance in the data. This assumption is based on the observation from Hi-C and ChIA-PET data analysis that true interactions occur within certain range of genomic span. Our goal is to identify complexes with one dominating distance between fragments. Using the probability vector of the neighboring distance, we quantify the likelihood of a dominating event. Formally, for an observed complex OC_*m*_ with *n* fragments and **p**_F2F_(OC_*m*_) = [*p*_1_, *p*_2_, …, *p*_*n* − 1_], we compute the normalized Shannon entropy (Shannon [27])

$$ {H}_{\mathrm{norm}}\left({\mathbf{p}}_{\mathrm{F}2\mathrm{F}}\left({\mathrm{OC}}_m\right)\right)=\frac{\sum_{i=1}^{n-1}{p}_i{\log}_2\left(\frac{1}{p_i}\right)}{\log_2\left(n-1\right)}. $$

The normalization factor log_2_(*n* − 1) ensures that *H*_norm_(***x***) ∈ [0, 1] for any probability vector ***x***. Generally, *H*_norm_ is small when only one or two of *p*_*i*_ of are large, in which case we presume that a complex is a multiplet and need to separate into singlets. For each observed complexes in the “deferred” category, we compare its normalized Shannon entropy to the average normalized Shannon entropy of the expected complexes in the corresponding class; if the former is smaller, then we separate the observed complex at the longest distance interaction. In other words, for OC_*m*_ ∈ *F*_*j*, def_, if

$$ {H}_{\mathrm{norm}}\left({\mathbf{p}}_{\mathrm{F}2\mathrm{F}}\left({\mathrm{OC}}_m\right)\right)<\frac{1}{\mathrm{100,000}}{\sum}_{k=1}^{\mathrm{100,000}}{H}_{\mathrm{norm}}\left({\mathbf{p}}_{\mathrm{F}2\mathrm{F}}\left({\mathrm{EC}}_k^j\right)\right), $$

then OC_*m*_ is separated into

OC_*m*, 1_= $\left\{{u}_m^1,{u}_m^2,\dots, {u}_m^S\right\}$ and OC_*m*, 2_ = $\left\{{u}_m^{S+1},{u}_m^{S+2},\dots, {u}_m^n\right\}$,

where $d\left({u}_m^S,{u}_m^{S+1}\right)=\max {\mathbf{x}}_{\mathrm{F}2\mathrm{F}}\left({\mathrm{OC}}_m\right)$. Furthermore, if the second largest distance is at least $\frac{1}{\tau }$ of the largest distance, we also separate at the second longest distance. *τ* is a variable parameter and we set it to 2 in our analyses; the larger the *τ*, the more likelihood of a “second cut” (implying a triplet). The resulting sub-complexes are placed in *F*_*j*, def, filt_ and are now subject to the second distance test. Note that we did not perform any statistical test in this step, only performed filtering. Also, the Shannon entropy merely serves as a quantification measure for a single complex and should not be confused with the heterogeneity of all complexes in the ChIA-Drop data.

#### Second distance test

We repeat the distance test after correcting for possible doublets and triplets. For a OC_*m*, ∗_ ∈ *F*_*j*, def, filt_

$$ \mathrm{pva}{\mathrm{l}}_{\mathrm{raw}}\left({\mathrm{OC}}_{m,\ast}\right)={\sum}_{k=1}^{\mathrm{100,000}}{1}_{d_{\mathrm{tot}}\left(\mathrm{O}{\mathrm{C}}_{m,\ast}\right)>{d}_{\mathrm{tot}}\left(\mathrm{E}{\mathrm{C}}_k^j\right)}. $$

We adjust raw *p* values using Benjamini-Hochberg method with false discovery rate (FDR) of 0.1. The complexes with adjusted *p* value ≤0.1 are classified as “pass2” (*F*_*j*, pass2_) and others are “fail2” (*F*_*j*, fail2_). A diagram of the distance test is illustrated in Additional file 1: Figure S1a.

#### Implementation, results, and analysis

MIA-Sig takes putative chromatin complexes as the input, which are results of the ChIA-DropBox (Tian et al. [29]) data processing and visualization pipeline. The “distance test” python (v3.6) script encompasses all parts using the following packages: numpy, random, statsmodels, itertools, os, and sys. We used the parameters --gen dm3 --fdr 0.1 --cef 2 --sz 100,000 to run the script on GSM3347523 dataset, which used 1.8 GB of memory and 13 min of CPU time. To save memory, we store minimal information for the null, total distance for expected complexes, and their mean entropy for each fragment class. Two runs with the same parameters should yield identical results because we set seeds in the construction step for the expected complexes. By saving the first 1000 expected complexes for each class in a chromosome, we can compare our expected null model to the biological null model, which is the “pure DNA” described in (Zheng et al. [31]). Plotting the neighboring distances, we observed that both the computational null and pure DNA are unimodal with peaks between 1 and 10 Mbps for all classes (Additional file 1: Figure S1b). After confirming that our expected complexes do emulate long-range noise, we obtained detailed statistics of each step resulting in 55,995 significant complexes (Additional file 1: Figure S1c). Complexes in each of the “original,” “significant” (“pass1” + “pass2”) and “insignificant” (“fail1” + “fail2”) categories are converted into a .short format by enumerating over all pairs of fragments in a complex. Three .short files are then converted into .hic files via juicer (v1.7.5) to be visualized in juicebox. A 5-Mbps window on chr3L shows that the original data exhibit both the signal and noise, which are separated by MIA-Sig into significant and insignificant, respectively (Additional file 1: Figure S2a). The original observed complexes have a bimodal distribution for high fragment classes, which is a distinct behavior from the null distribution (Additional file 1: Figure S1b, S2b). The density plot further supports that significant complexes retained short distances or a mix of short and long distances. By contrast, insignificant complexes are only comprised of unimodal long distances (Additional file 1: Figure S2b). Consistent with an observation that high-fragment complexes contribute to the structure more than the low-fragment complexes (Zheng et al. [31]), MIA-Sig assessed the majority of high-fragment complexes as significant (Additional file 1: Figure S2c). We next investigated the effects of the entropy filter, which was designed to remove doublets and triplets. Of the 1,452,878 complexes in the deferred category ranging from *n* = 3 to *n* = 8, MIA-Sig identified 60% (869,065) to be singlets, 34% (498,291) to be doublets, and 6% (85,522) to be triplets, yielding 548,342 singletons (*F*_1_) and 1,573,871 complexes (*F*_≥2_) (Additional file 1: Figure S3). For each class, singletons had the highest normalized Shannon entropy, followed by doublets and triplets. The entropy filter step allowed MIA-Sig to identify additional 15,055 complexes as significant, which amounts to 27% of the total significant complexes.

### TAD calling for non-enriched multiplex chromatin interactions data

#### Generating 1D signal track

Existing TAD calling algorithms for pairwise Hi-C data generally fall into two categories: (1) signal segmentation after conversion from 2D contact maps into 1D tracks measuring interaction intensities along the genome and (2) community detection directly on the 2D heatmap by treating each bin as a node on an undirected graph. We take the first approach and convert our complexes into 1D signal track. A conventional pairwise approach would enumerate over all pairs of fragments in a complex and record their spans. However, multi-fragment complexes may over-contribute since the number of pairs grows quadratically: $\left(\genfrac{}{}{0pt}{}{n}{2}\right)=\frac{n\left(n-1\right)}{2}$, where *n* is the number of fragments in a complex. Instead, we allow each complex to only contribute linearly in *n* by recording its span weighted by *n*. More precisely, coordinates are (chrom(${u}_m^1\Big)$, start(${u}_m^1$), end(${u}_m^n$), *n*) for an OC_*m*_ with *n* fragments. We finally obtain a “weighted complex span coverage” by accumulating the coordinates over all given complexes.

#### Smoothing and segmentation

Our next task is to segment the 1D track into regions with a high signal and annotate them as TADs. In an ideal case, we can achieve this goal by computing the slope of the signal **s** and by recording critical points where the slope is 0. However, our signal has a basepair resolution and thus is not smooth, resulting in too many false critical points. A common way to smooth the signal is by a moving average window, but using a large window size would lose the resolution and yield TADs with fuzzy boundaries. Moreover, due to the inherent nature of TAD sizes, a window size parameter optimal in one region may not be optimal in another. We avoid this parameter tuning step by instead applying a discrete wavelet transformation, which decomposes signal into high-frequency component and low-frequency component (Mallat [19]). Of note, the low-frequency component generally retains the smoothed version of the signal without affecting the shape, which is helpful for us to find accurate TAD boundaries (Additional file 1: Figure S4). Using this “smoothed” signal, we compute the slope and fine-tune TAD coordinates.

#### Implementation, results, and analysis

The “tad calling” python (v3.6) script encompasses all parts using the following packages: numpy, os, scipy, pywt, itertools, and sys. We used the parameters --cat PASS --bs 1000 –sp drosophila --r dm3 to run the script on significant complexes from the distance test of GSM3347523 dataset, which used 84 MB of memory and 1 min of CPU time. Before generating the 1D signal track, we separate 2 fragments if they are more than 100 Kb apart, based on the upper range of general TAD sizes by organisms (Dekker and Heard [6]). Coverage was generated by BEDtools (Quinlan and Hall [24]) using “genomecov” function, and the coverage is binned into 1-Kb windows via “makewindows” and “map” commands. Signal smoothing was done by pywt package using the parameters “bior1.1” for wavelet function and “3” for the level. MIA-Sig called 335 TADs over the 6 chromosomes, with a median size of 200 Kb (Additional file 1: Figure S5a). For a comparison, we also tested insulation score as follows: .hic file (of all pairs of fragments) are converted into contact matrices via Juicer’s “dump” function with a dense matrix option (-d) in the Juicer tool (v1.7.5); insulation score script (https://github.com/dekkerlab/cworld-dekker/tree/master/scripts/perl) is executed with 100-Kb insulation square size, 100-Kb delta window size for 10-Kb resolution contact maps with balanced normalization. Insulation score (InS) called 513 TADs with a median of 150 Kb and did not call any TADs larger than 500 Kb (Additional file 1: Figure S5b,c). When we examined the gaps (defined as the regions between 2 TADs, if any), MIA-Sig also had a wider size range than InS (Additional file 1: Figure S5d,e). For each TAD called by MIA-Sig and InS, we compute the total H3K27me3 signal and plot the genome-wide behavior (Additional file 1: Figure S6a). Overall, MIA-Sig has a higher inactive signal in TADs than InS. The gap regions in *Drosophila* are known to be transcriptionally active and should positively correlate with the H3K27ac signal. We confirm that MIA-Sig has a slightly higher median active signal than InS (Additional file 1: Figure S6b). Note that we did not perform any normalization by region size because both algorithms segment the genome into either a TAD or a gap, so the region size should also be a feature. Histone marks provide biological evidence that MIA-Sig TADs are inactive and gaps are active, but ChIA-Drop fragment counts provide a direct measure of TAD and gap intensities. Using the BEDtools command “intersect -c,” we count the number of fragments in each region. MIA-Sig generally captured more fragments in TADs than InS did (Additional file 1: Figure S6c) and less fragments in gaps than InS (Additional file 1: Figure S6d). Finally, we annotate each fragment in significant and insignificant complexes as “TAD” or “gap” as called by MIA-Sig. For each complex, we count the number of TADs with at least 2 fragments within each TAD. Only 5% of the insignificant complexes had fragments in 1 or 2 TADs, and the rest were not contributing to the TAD structure (Additional file 1: Figure S7a), validating the observation from 2D heatmaps. By contrast, only 26% of the significant complexes were not in TADs, a majority (51.3%) in intra-TAD interactions, and many (23%) connected 2 or more TADs. By observing that 12,884 complexes involve 2 to 21 TADs, we next sought to characterize if multiple complexes connect the same set of TADs.

### Inter-TAD binomial test for non-enriched multiplex chromatin interaction data

#### Motivation and intuition

Our goal is to evaluate the statistical significance of these TAD combinations based on the frequency of occurrence measured by the number of complexes therein. The problem is simple for a pair of TADs: we may treat a TAD as a ChIA-PET loop anchor and apply tools based on the hypergeometric test. However, our data are now multi-dimensional. For instance, suppose that there are five TADs and five combinations “A-C,” “B-C,” “B-C-D,” “A-B-E,” and “A-D-E” (Additional file 1: Figure S7b). The pair “B-C” appears four times on its own, but also appears three times as a part of the triple “B-C-D.” Moreover, some parts of a combination may appear elsewhere with the same number of TADs: given “B-C-D” and “A-C-D,” “C-D” appears twice. Therefore, we propose a counting scheme based on the occurrence of “expanded pairs.”

#### Methods

The notations used defined in this section are independent from those in other sections. We let the *i*th combination be ${\mathrm{TC}}_i=\left\{{T}_i^1,{T}_i^2,\dots, {T}_i^N\right\}$, where each ${T}_i^n\in \left\{{\mathrm{TAD}}_1,{\mathrm{TAD}}_2,\dots, {\mathrm{TAD}}_M\right\}$, *N* =  ∣ TC_*i*_∣ is the number of TADs involved, and we partition each TC_*i*_ into the same class *G*_*j*_ if |TC_*i*_| = *j*. All pairs of TADs in *TC*_*i*_ are in Pa(TC_*i*_) = {{*r*, *s*} : *r* ≠ *s*, for *r*, *s* ∈ TC_*i*_} and $\left|\mathrm{Pa}\left(\mathrm{T}{\mathrm{C}}_i\right)\right|=\frac{n\left(n-1\right)}{2}$. For each TC_*i*_, we record the number of pairs in the same class as

$$ a\left(\mathrm{T}{\mathrm{C}}_i\right)=\sum \limits_{y\in {G}_N}\sum \limits_{w\in \mathrm{Pa}(y)}{1}_{w=\mathrm{Pa}\left(\mathrm{T}{\mathrm{C}}_i\right)} $$

and the number of exact appearance in higher class as

$$ b\left(\mathrm{T}{\mathrm{C}}_i\right)={\sum}_{w\notin {G}_N}{1}_{\mathrm{T}{\mathrm{C}}_i\subset w}. $$

Using these two numbers, we compute the appearance of “pairs” in the same class and higher class

$$ x\left({\mathrm{TC}}_i\right)=a\left(\mathrm{T}{\mathrm{C}}_i\right)+b\left(\mathrm{T}{\mathrm{C}}_i\right)\bullet n\left(\frac{n-1}{2}\right). $$

Finally, we perform the binomial test with *x*(TC_*i*_) as the number of success, $k\left(\mathrm{T}{\mathrm{C}}_i\right)=\sum \limits_{z\in {G}_j}x(z)$ as the number of trials, the probability of success hypothesized as $p=\frac{1}{\mid {G}_j\mid }$; the alternative hypothesis is that the observed probability is greater than the expected probability *p*. A detailed example is provided using the same notations (Additional file 1: Figure S7b).

#### Implementation, results, and analysis

A python script “inter-TAD binomial test” implements the method using packages numpy, itertools, scipy, statsmodels, os, and sys. Of 6861 unique combinations involving 2 to 21 TADs, 915 (13%) were identified as statistically significant. An example illustrates that a pair of TADs with a strong signal in the heatmap and many complexes in the linear view has lower *p* value than that with a weak signal (Additional file 1: Figure S7c). Here, we assumed that the frequency of interactions between TADs is independent of their distance and sizes, and we also did not distinguish contacts with 2 fragments from those with 10 fragments. These parameters may be incorporated in the future version.

### Enrichment test for RNAPII-enriched multiplex chromatin interaction data

#### Motivation

The above sections are designed to analyze non-specific multiplex interaction data analogous to the Hi-C data. With an additional step of chromatin immunoprecipitation, protein-enriched multiplex data reveal protein-specific interactions similar to the population average ChIA-PET loops. In a typical ChIA-PET analysis, loops anchored in strong binding peaks are considered to be more reliable than those with weak or no peaks. Extending this notion to the multiplex data, we developed an enrichment test for RNAPII ChIA-Drop data. Our end goal is to retain complexes with fragments in strong binding peaks. One approach is to call peaks and only keep complexes that overlap the peak regions. However, peak calling algorithms have their own model assumptions that may not hold for ChIA-Drop data. Even with accurate peak regions, assigning statistical significance to each complex is not a trivial problem since the null distribution is unclear. Thus, we take an alternative—inevitably the computationally expensive—approach by sampling the background null distribution for each complex.

#### Statistical test

The idea is to take the observed complex and place it on a random location of the same chromosome and compare the mean coverage between the observed and the expected. Through many rounds of re-sampling, we obtain the *p* value by counting the number of occurrences in which the expected coverage exceeds the observed coverage (Additional file 1: Figure S8a). More precisely, for an observed complex ${\mathrm{OC}}_m=\left\{{u}_m^1,{u}_m^2,\dots, {u}_m^n\right\}$, we randomly draw an integer $i\in \left\{1,\dots, \mathrm{length}\left(\mathrm{chrom}\right)-\mathrm{start}\left({u}_m^1\right)\right\}$ and the shift $\delta =\mathrm{start}\left({u}_m^1\right)-i$. The first expected complex is then ${\mathrm{EC}}_1^m=\left\{{v}_m^1,{v}_m^2,\dots, {v}_m^n\right\}$, where $\mathrm{start}\left({v}_m^l\right)=\mathrm{start}\left({u}_m^l\right)-\delta$, and $\mathrm{end}\left({v}_m^l\right)=\mathrm{end}\left({u}_m^l\right)-\delta$ for all *l* ∈ {1, …, *n*}. Repeating this process 10,000 times, we obtain $\mathrm{E}{\mathrm{C}}_k^m$ for *k* ∈ {1, …, 10,000}. We can then compute the raw *p* value of the *m*th observed complex as:

$$ \mathrm{pva}{\mathrm{l}}_{\mathrm{raw}}\left({\mathrm{OC}}_m\right)={\sum}_{k=1}^{\mathrm{10,000}}{1}_{\mathrm{covg}\left(\mathrm{O}{\mathrm{C}}_m\right)<\mathrm{covg}\left(\mathrm{E}{\mathrm{C}}_k^m\right)}, $$

where $\mathrm{covg}\left(\mathrm{O}{\mathrm{C}}_m\right)=\frac{1}{n}{\sum}_{l=1}^n\frac{\boldsymbol{fcs}\left(\mathrm{start}\left({u}_m^l\right),\mathrm{end}\left({u}_m^l\right)\right)}{\mathrm{end}\left({u}_m^l\right)-\mathrm{start}\left({u}_m^l\right)}$ and ***fcs***(*x*, *y*) is the mean “fragment coverage signal” between coordinates *x* and *y*. Raw *p* values are separated by chromosomes and are adjusted via the Benjamini-Hochberg method with a false discovery rate (FDR) of 0.1. The complexes with adjusted *p* value ≤0.1 are considered to be statistically significant and are classified as “pass”; others are considered insignificant or “fail.”

#### Implementation, results, and analysis

A python script “enrichment test” utilizes the packages numpy, random, statsmodels, os, and sys. GSM3347525 RNAPII ChIA-Drop data are pre-processed to exclude fragments mapped to the repetitive regions in the genome (dm3.rmsk.bed), and 769,803 complexes remain as “GSM3347525NR.” The most time-consuming part of the algorithm is to obtain the fragment coverage at a given location, since we need to search for a start and end indexes in a bedgraph or a bigwig file. With at least 769,803 × 2 × 10,000 = 1.54 × 10^10^ operations, we realized that python implementations of exact search would be intractable. As means to reduce the runtime, we store the bedgraph file into bins of size 10 bp and store only the fourth column “value.” The solution then turns into a simple lookup operation, yielding an approximation that is close to the exact solution. Our code is “parallelized” by chromosome, each using around 5 h CPU time and 230 MB of memory (Additional file 1: Figure S8b). MIA-Sig identified 190,226 complexes (24.7%) as statistically significant. We ensure that our empirical null distribution does behave randomly by comparing the enrichment scores of the observed complexes in chr2L with those of 1000 expected complexes generated for each observed complex (Additional file 1: Figure S8c). Zooming in further, we note that the histogram of the observed is shifted to the right of the histogram of the expected null (Additional file 1: Figure S8d). Using the active and inactive regions defined in (Zheng et al. [31]), we count the number of fragments therein for significant and insignificant complexes (Additional file 1: Figure S9a). For each active and inactive region, we compute the number of significant complex fragments and their log10 values are plotted (Additional file 1: Figure S9b); K-S test supports that significant complexes are indeed more likely to be in active regions than in inactive regions. By contrast, insignificant complexes have no bias towards or against active regions (Additional file 1: Figure S9c). We define a gene promoter as ± 1 KB from the transcriptional start site (TSS) annotated by UCSC genome browser. Note that typically ± 250 bp is used for *Drosophila*, but we extend it to accommodate ChIA-Drop protocol-specific features. A gene is active (6466 genes) if the total RNA-seq level is greater than 5 and inactive (8874 genes) otherwise. A fragment is “active promoter” if it overlaps the promoter of an active gene. In general, significant complexes have higher proportion of promoter fragments than insignificant complexes (Additional file 1: Figure S9d), and the skew is more pronounced for active promoters (Additional file 1: Figure S9e). Inactive promoters serve as a control, in which both significant and insignificant complexes display similar patterns in the number of inactive promoter fragments (Additional file 1: Figure S9f,g).

### Distance test on mouse F121 SPRITE data

We have performed the distance test on SPRITE data (Quinodoz et al. [25]) generated from F121 mouse embryonic stem cells (GSE114242) mapped to the mm9 reference genome. The pre-processing steps of the SPRITE data are the following: (1) extract complexes in chr18, (2) construct fragments by extending a read mapped position by 1000 bp, (3) exclude read mapped position if it is less than 10,000 bp away from the left-adjacent mapped loci, and (4) only retain complexes with 2 to 500 fragments. These parameters are chosen because Quinodoz et al. treat multiple reads in a bin to be 1 read due to PCR duplicates, where the bin sizes are 10 kb, 20 kb, 25 kb, 40 kb, 50 kb, 200 kb, 250 kb, and 1 Mbps. After converting reads into fragments in our standard file format of 1 complex per line, we ran the distance test with the parameters --gen mm9 --fdr 0.1 --cef 2 --sz 10,000. One modification is that during the first distance test, we “fail” the complexes with more than 100 fragments. The resulting master file is used for generating the 2D contact maps for ALL and PASS categories by enumerating all pairs of fragments in a complex (Fig. 3b). Likewise, the empirical cumulative distribution function is plotted for ALL and PASS categories (Fig. 3c).

## Supplementary information

**Additional file 1: Figure S1.** Overview of distance test, comparison of computational and experimental null distribution, and summary statistics. **Figure S2.** Characteristics of original, significant, and insignificant complexes. **Figure S3.** Effects of the entropy filter. **Figure S4.** Ideas behind the MIA-Sig TAD calling algorithm. **Figure S5.** Statistics of TAD and gap sizes called by MIA-Sig and Insulation Score. **Figure S6.** Comparison of TADs and gaps by MIA-Sig and Insulation Score. **Figure S7.** Inter-TAD binomial test. **Figure S8.** Overview of enrichment test for RNAPII ChIA-Drop data. **Figure S9.** Comparison of significant and insignificant RNAPII complexes. **Figure S10.** Annotation of fragment as an active promoter, inactive promoter, or non-promoter.

**Additional file 2.** Review history.

## Data Availability

The MIA-Sig software is available under the MIT License at GitHub [12]. A version of the source code used in this manuscript is deposited on Zenodo [13]. ChIA-Drop (GSM6647523) and RNAPII ChIA-Drop (GSM3347525) data were downloaded from the Gene Expression Omnibus (GEO) under SuperSeries accession number GSE109355 [31]. SPRITE “mouse_combined_clusters” data were downloaded from the GEO under SuperSeries accession number GSE114242 [25]. A link to the pure DNA ChIA-Drop data and processed files of relevant data is also available through the MIA-Sig GitHub page [12].
