# Supplementary material for: MIA-Sig: multiplex chromatin interaction analysis by signal processing and statistical algorithms
Source: Genome Biol. 2019 Nov 25;20:251. doi: 10.1186/s13059-019-1868-z (PMC6876102; doi:10.1186/s13059-019-1868-z)
Supplement: Supplementary file 1 — Additional file 1: Figure S1. Overview of distance test, comparison of computational and experimental null distribution, and summary statistics. Figure S2. Characteristics of original, significant, and insignificant complexes. Figure S3. Effects of the entropy filter. Figure S4. Ideas behind the MIA-Sig TAD calling algorithm. Figure S5. Statistics of TAD and gap sizes called by MIA-Sig and Insulation Score. Figure S6. Comparison of TADs and gaps by MIA-Sig and Insulation Score. Figure S7. Inter-TAD binomial test. Figure S8. Overview of enrichment test for RNAPII ChIA-Drop data. Figure S9. Comparison of significant and insignificant RNAPII complexes. Figure S10. Annotation of fragment as an active promoter, inactive promoter, or non-promoter. [file 13059_2019_1868_MOESM1_ESM.docx]

Review History

**First round of review**

**Reviewer 1**

**Were you able to assess all statistics in the manuscript, including the appropriateness of statistical tests used?**

No

**Were you able to directly test the methods?**

No

**Comments to author:**

In this manuscript, Kim et al present a method for multiplex chromatin interaction analysis. There are several new methods for multiplex chromatin interactions, one of which is ChIA-drop, which several of these authors have pioneered. These methods offer a new window into the formation of multiplex higher-order chromatin interactions. However, there are no methods currently available for multiplex chromatin interaction library analysis. Therefore, Kim et al's method fills a sorely-needed gap in the field. The figures are well prepared, however, I have several major concerns that should be addressed.

1. MIA-Sig can identify not only the multiplex chromatin interactions but also traditional chromatin interactions. How does it perform on traditional chromatin interactions and TADs? How is this in comparison with other methods for studying chromatin interactions and TADs? Have any of the chromatin interactions and TADs that can be found by MIA-Sig but not by other methods for calling chromatin interactions been validated?

2. Given that MIA-Sig is presented as a method for studying multiplex chromatin interactions, it should be applied not just to ChIA-Drop but also to one of the other multiplex methods such as GAM or SPRITE, to demonstrate that this method is indeed useful for such other datasets.

3. Several parts of the manuscript are very dense and difficult to understand especially for someone who may not be in the chromatin interaction field, and not a bioinformatician. The entire manuscript should be proofread, and concepts such as "distance test" and "entropy filter" (shown, for example, in Figure S1), and the difference between "experimental null" and "computational null" can be explained in more detail in the figure legend to better help the readers to understand. For figure 1, it is unclear if the results shown are for human or drosophila ChIA-drop. For the discussion it would be helpful if the significance of the work is more clearly explained, e.g. the section on "Wavelet-based segmentation method identifies TADs overlapping inactive regions" - what is the significance of this finding? Does it show that the wavelet-based segmentation method is better in some way? Or does it show new biology, and in this case are there any complementary approaches for confirming this finding?

**Reviewer 2**

**Were you able to assess all statistics in the manuscript, including the appropriateness of statistical tests used?**

Yes: All statistical tests in the manuscript seem appropriate to the best of my knowledge.

**Were you able to directly test the methods?**

Yes.

**Comments to author:**

The manuscript by Kim et al. describes a computational method for de-noising and analyzing multiplex chromatin interaction data generated from techniques such as ChIA-Drop. The MIA-Sig method includes several useful components designed for ChIA-Drop data, and can be extended to other multiplex chromatin interaction data. Having comprehensive data processing and analysis features, MIA-Sig can be very helpful to the chromatin interaction data analysis and is of broad interest to the 3D genome research community.

The method is well designed, and the analyses are clearly described overall. The authors can consider several comments listed below in order to further improve the work so that the manuscript can be accepted for publication in Genome Biology.

1. The distance test using Shannon entropy is a novel approach to identify multiplets and separate doublets to singlets. This is useful in both quality assessment and data analysis. However, the authors did not describe the specificity of this approach. There could be real complexes discarded by the current approach, using the seemingly arbitrary cutoffs. A systematic evaluation of the cutoff determination can better justify the model and increase the utility of the method.

2. A few data analytics and visualization features that were used to generate some results and plots in the manuscript (e.g., Figs 1c, 2f, Suppl. Figs S3, S8, and S9) can be incorporated in the MIA-Sig software package, so that MIA-Sig can serve as a comprehensive pipeline including both data QC and data analysis for ChIA-Drop and other multiplex chromatin interaction assays. In this way other researchers can use MIA-Sig to conduct more data analysis easily.

**Authors Response**

**Point-by-point responses to the reviewers’ comments:**

POINT BY POINT RESPONSE TO REFEREES’ COMMENTS

Manuscript ID: [GBIO-D-19-01246]

We thank the reviewers for their constructive feedback, which has helped us improve our manuscript. Following the reviewers’ suggestions, we have applied the distance test on SPRITE data, clarified the text, benchmarked parameters, and included scripts to generate useful plots. We believe the revision has greatly improved the quality of the manuscript. In addition to the revised manuscript, our GitHub repository is updated and our code is now on zenodo.

Please find our point-by-point responses to the reviewers’ comments below. We have formatted the reviewers’ comments in italic blue while our responses are in black. To facilitate our responses, we have included supporting figures in this document.

Reviewer 1

In this manuscript, Kim et al present a method for multiplex chromatin interaction analysis. There are several new methods for multiplex chromatin interactions, one of which is ChIA-drop, which several of these authors have pioneered. These methods offer a new window into the formation of multiplex higher-order chromatin interactions. However, there are no methods currently available for multiplex chromatin interaction library analysis. Therefore, Kim et al's method fills a sorely-needed gap in the field. The figures are well prepared, however, I have several major concerns that should be addressed.

*We thank the reviewer for recognizing the value of the method to the field. Our revised manuscript includes additional results and clarifications to address the reviewer’s concerns.*

Detailed review:

MIA-Sig can identify not only the multiplex chromatin interactions but also traditional chromatin interactions. How does it perform on traditional chromatin interactions and TADs? How is this in comparison with other methods for studying chromatin interactions and TADs? Have any of the chromatin interactions and TADs that can be found by MIA-Sig but not by other methods for calling chromatin interactions been validated?

*We appreciate the reviewer’s recognition that MIA-sig could also be used for traditional chromatin interaction data. However, the initial version of MIA-Sig was intended to be applicable only to multiplex chromatin interactions data.*

*It is possible for us to modify and test scripts in MIA-Sig for analyzing traditional chromatin interactions. However, considering that there are already many tools available for analyzing Hi-C and ChIA-PET data, whereas there is a lack of tools for SPRITE, GAM, and ChIA-Drop data analysis, our immediate priority has been to refine its functionality for multiplex data.*

*Regarding the last part of the reviewer’s question, the main goal of MIA-Sig was to call quality multiplex chromatin interactions (≥ 3 fragments per chromatin complex) that could not be assessed by the methods designed for calling of pairwise contacts from “traditional chromatin interactions.” The most important function of MIA-Sig is to denoise and assess statistical significance of multiplex chromatin interactions, and TAD analyses are of secondary priority.*

*To formalize our intention, we start the discussion section with the following paragraph:*

*“Many tools exist for analyzing traditional proximity ligation-based chromatin interaction data, such as Hi-C and ChIA-PET. By contrast, there is a lack of tools to comprehend the data generated by the recently developed multiplex interaction mapping techniques. We have developed MIA-Sig to fill in this gap in the latter category and intend it to be applicable only to multiplex chromatin interaction data.”*

2) Given that MIA-Sig is presented as a method for studying multiplex chromatin interactions, it should be applied not just to ChIA-Drop but also to one of the other multiplex methods such as GAM or SPRITE, to demonstrate that this method is indeed useful for such other datasets.

*The reviewer’s point is valid. GAM data are generated by taking one physical slice per nucleus. As a result, the data are stored as a unit of nucleus and may not capture one chromatin complex in a similar way that SPRITE and ChIA-Drop do. By contrast, SPRITE uses 3 to 5 rounds of split-and-pool approach to barcode each chromatin complex and thus reads with the same unique barcode represent genomic loci involved in one chromatin complex. Therefore, in theory, the SPRITE data should be comparable to the ChIA-Drop data, and be applicable to MIA-Sig for analysis.*

*We have now performed the distance test on SPRITE data (Quinodoz et al., Cell, 2018) generated from F121 mouse embryonic stem cells (GSE114242). Detailed processing steps are provided in the “Distance test on mouse F121 SPRITE data” of the Methods section in our revised manuscript. The results are shown for chr18 in Figure 4, where the original complexes exhibit off-diagonal noise and significant (‘pass’) complexes have the majority of the signal along the diagonal (panel b). The empirical cumulative distribution function show that the neighboring distances for the significant complexes are shorter than those for the original complexes (panel c), supporting the observation in 2D contact maps. Full results are reported in the “Distance test can be applied to SPRITE data” of the Results section.*

*
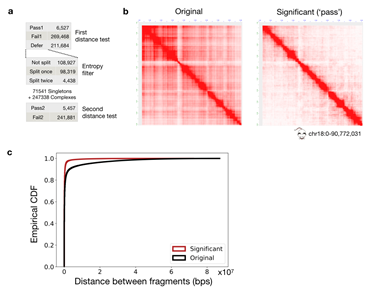
*

*Figure 3: Distance test results on SPRITE mouse embryonic stem cell dataset.*

*(a) Of 487,679 complexes in chr18, 11,984 (2.46%) complexes pass by the two distance tests. (b) A chromosome-wide heatmap is generated for original and significant complexes by enumerating all pairs of fragments. (c) Empirical cumulative distributive function (ECDF) for the neighboring distances of original and significant complexes (two-sided Kolmogorov-Smirnov test statistic=0.18, p-value <2.2×10^(-16) ).*

3) Several parts of the manuscript are very dense and difficult to understand especially for someone who may not be in the chromatin interaction field, and not a bioinformatician. The entire manuscript should be proofread, and concepts such as "distance test" and "entropy filter" (shown, for example, in Figure S1), and the difference between "experimental null" and "computational null" can be explained in more detail in the figure legend to better help the readers to understand. For figure 1, it is unclear if the results shown are for human or drosophila ChIA-drop. For the discussion it would be helpful if the significance of the work is more clearly explained, e.g. the section on "Wavelet-based segmentation method identifies TADs overlapping inactive regions" - what is the significance of this finding? Does it show that the wavelet-based segmentation method is better in some way? Or does it show new biology, and in this case are there any complementary approaches for confirming this finding?

*We acknowledge the reviewer’s critism on our writing style and revised the manuscript accordingly for the general readers of Genome Biology. Please find the following changes:*

- *Explanations of “distance test”, “entropy filter”, “experimental null” and “computational null”*
  - *Figure S1 legend*

*Supplementary Figure S1: Overview of distance test, comparison of computational and experimental null distribution, and summary statistics.*

*(a) A diagram of distance test encompasses both the statistical test and the filter for correcting multiplets, which are defined as droplets with more than one chromatin complex. All observed complexes get tested for distance, and those with significant p-values (short distances) ‘pass’; of those with insignificant p-values (large distances), 2-fragment complexes ‘fail’, and others are ‘deferred’. The complexes in the ‘deferred’ category may include multiplets, which are separated into multiplets by the Entropy filter. An intuition is that the fragment-to-fragment distances are approximately uniform for singlets (OC2) and include one large distance for doublets (OC5). The resulting sub-complexes are subject to the second distance test. (b) Log10 of neighboring fragment-to-fragment distances of complexes are computed by fragment number class. The expected complexes are computationally constructed by randomly selecting distances from observed complexes, whereas the pure DNA complexes are not chromatin-associated and hence lack structures. Density plot illustrate that both datasets comprise mostly large distances.*

- *Clarifications of the species in the experiments*
  - *Figure 1 legend: Performance of MIA-Sig on Drosophila S2 cells ChIA-Drop data.*
  - *Figure 2 legend: Enrichment test on Drosophila S2 cells RNAPII ChIA-Drop data.*
  - *All genome browser screenshots now have either a fruitfly icon (for Drosophila S2 cells ChIA-Drop data) or a mouse icon (for Mouse F121 cells SPRITE data)*
- *Highlighting the significance of the work in the discussion section*

Reviewer 2

The manuscript by Kim et al. describes a computational method for de-noising and analyzing multiplex chromatin interaction data generated from techniques such as ChIA-Drop. The MIA-Sig method includes several useful components designed for ChIA-Drop data, and can be extended to other multiplex chromatin interaction data. Having comprehensive data processing and analysis features, MIA-Sig can be very helpful to the chromatin interaction data analysis and is of broad interest to the 3D genome research community.

The method is well designed, and the analyses are clearly described overall. The authors can consider several comments listed below in order to further improve the work so that the manuscript can be accepted for publication in Genome Biology.

*We appreciate the reviewer’s encouraging comments and constructive feedback, which prompted us to clarify the descriptions of the distance test and to update our software package.*

The paper would be stronger if the authors could address the following points:

1) The distance test using Shannon entropy is a novel approach to identify multiplets and separate doublets to singlets. This is useful in both quality assessment and data analysis. However, the authors did not describe the specificity of this approach. There could be real complexes discarded by the current approach, using the seemingly arbitrary cutoffs. A systematic evaluation of the cutoff determination can better justify the model and increase the utility of the method.

*We agree with the reviewer that the cut-off threshold for the entropy filter seems arbitrary at a glance. However, we had stated the following in the Methods section: “For each observed complexes in the ‘deferred’ category, we compare its normalized Shannon entropy to the average normalized Shannon entropy of the expected complexes in the corresponding class; if the former is smaller, then we separate the observed complex at the longest distance interaction.” Here, the expected complexes are constructed from the resampling-based null model, which is shown to be similar to the experimental null in our dataset (Supplementary Figure S1b).*

*To minimize any confusions, we introduce the idea in the manuscript in the earlier than the methods:*

*Results section*

*“The cut-off threshold is determined by the average normalized Shannon entropy of the expected null distribution as described below.”*

*Figure 1b legend*

*“(b) MIA-Sig aims to detect multiplets by computing the normalized Shannon entropy H_norm (Methods). It separates a complex at the largest distance if H_norm is smaller than a threshold, which is 0.7 in this example. This threshold is defined as the average normalized Shannon entropy of the expected null model.”*

*Supplementary Figure 3c (added)*


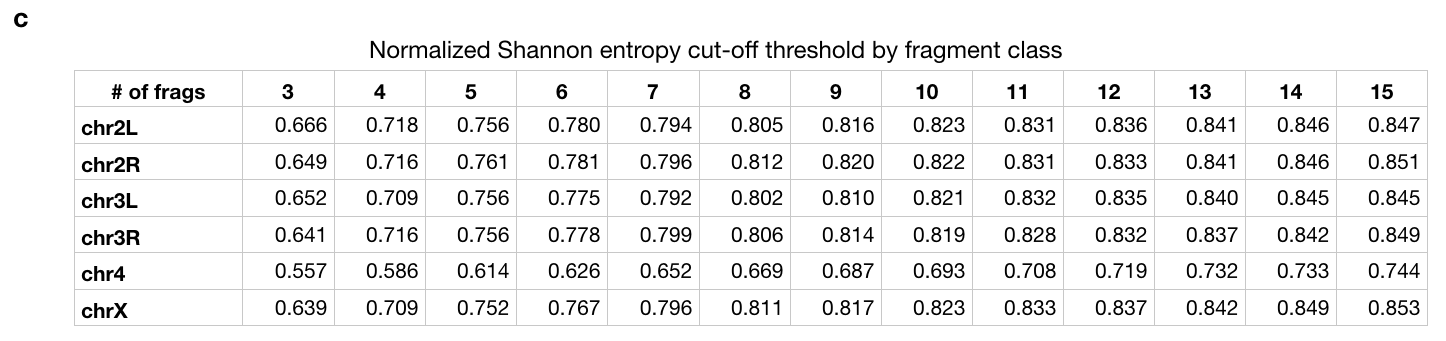


*Supplementary Figure 3c legend*

*“(c) The average normalized Shannon entropy of 1000 expected complexes (null) is recorded for each fragment class in a given chromosome. These values are used as a cut-off threshold in the ‘entropy filter’ step.”*

*Despite our reasonings and justifications of the null model, it is true that there could still be real complexes discarded by the current approach. As the notion of “real complexes” is not yet established, we evaluate our result by performing the K-S test on distance distributions between original and significant complexes; the statistic and p-values are recorded. We now include benchmark results by testing FDR=0.01, 0.05, 0.1, 0.2, 0.3, ratiothreshold=2,3,5, and samplesize=100000.*

*Supplementary Figure 3d (added)*

*
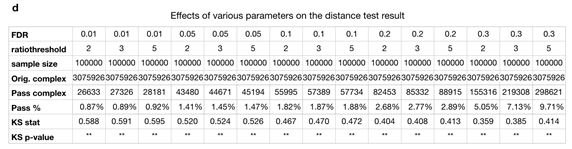
*

*Supplementary Figure 3d legend*

*(d) The effects of parameter selection in the distance test are evaluated by the two-sided K-S test statistics and p-value for the fragment-to-fragment distances of original and significant (pass) complexes. ** denotes p-value <2.2×10^(-16).*

2) A few data analytics and visualization features that were used to generate some results and plots in the manuscript (e.g., Figs 1c, 2f, Suppl. Figs S3, S8, and S9) can be incorporated in the MIA-Sig software package, so that MIA-Sig can serve as a comprehensive pipeline including both data QC and data analysis for ChIA-Drop and other multiplex chromatin interaction assays. In this way other researchers can use MIA-Sig to conduct more data analysis easily.

*We thank the reviewer for this suggestion. Our MIA-Sig software package now contains scripts to generate Figure 1e, Supplementary Figures S1b, S2bc, S3ab, S8c, and S8d. Figures 2f and Supplementary Figure S9 involve external datasets such as H3K27me3 and H3K27ac ChIP-seq, RNA-seq, and annotation of promoter regions according to user’s own definitions for each reference genome. Since these datasets may not be available for general users of MIA-Sig, we concluded that it is best to exclude them from the standard package and instead provided the scripts on a public Box Sync folder for researchers who may find the scripts to be useful.*

*Here are the changes to our codes:*

- *GitHub: https://github.com/TheJacksonLaboratory/mia-sig/wiki*
  - *Now includes scripts plot_dist_test_results.py and plot_enrich_test_results.py to generate figures that are generalizable and directly pertinent to MIA-Sig results (e.g., Figures 1e, S1b, S2bc, S3ab, S8c, S8d)*
- *Box Sync: https://thejacksonlaboratory.box.com/s/hmj9p227q22g91iemw3k7o1i655xeghn*
  - *Includes two optional python scripts (GSM3347523_figure_script.ipynb, GSM3347525N_figure_script.ipynb) that can reproduce all figures in this manuscript*

**Second round of review**

**Reviewer 1**

All my comments have been addressed.

**Reviewer 2**

The authors have satisfactorily addressed both of my questions.
